# Rosmarinic acid mitigates age-related loss of EEG complexity in temporal cortex: a recurrence quantification analysis

**DOI:** 10.1007/s11571-026-10457-9

**Published:** 2026-05-07

**Authors:** Serkan Uslu, Deniz Kantar

**Affiliations:** 1 Faculty of Medicine, Department of Biophysics, Akdeniz University, Antalya, Turkey; 2 Faculty of Medicine, Department of Biophysics, Bursa Uludağ University, Bursa, Turkey; 3 Dumlupınar Bulvarı, Tıp Fakültesi, Akdeniz Üniversitesi Kampüsü, 07070 Antalya, Turkey

**Keywords:** Aging, Electroencephalography (EEG), Entropy, Nonlinear dynamics, Rosmarinic acid, Neuroprotective agents

## Abstract

Physiological aging is associated with progressive changes in brain function, including neural oscillations. Recurrence Quantification Analysis (RQA) may provide a nuanced perspective on neural dynamics in aging. Rosmarinic acid (RA) has shown promise in mitigating age-related neurodegeneration. Its effects on EEG complexity in aging models remain unexplored. This study aimed to investigate age-related changes in RQA metrics and to assess whether RA modulates these alterations in a rodent aging model. Aging was induced in female rats via D-galactose administration. RA was administered to aged model animals. Urethane-induced EEG were obtained and analyzed across delta and theta bands. RQA parameters—determinism (DET), entropy (ENTR), and laminarity (LAM)—were computed for frontal and temporal regions. Aging was associated with a significant increase in DET and LAM, particularly in the temporal cortex, indicating enhanced regularity and persistence of EEG patterns. Concurrently, ENTR values declined, suggesting reduced signal complexity. RA partially reversed these trends, notably decreasing DET and LAM while increasing ENTR values in the temporal cortex. No significant changes were observed in the frontal cortex. This study underscores the utility of RQA in capturing nonlinear EEG alterations associated with aging and highlights RA as a promising compound for preserving cortical dynamics in senescence.

**Supplementary Information:**

The online version contains supplementary material available at 10.1007/s11571-026-10457-9.

## Introduction

Aging is a complex biological process marked by a progressive decline in physiological functions, including significant alterations in brain activity patterns. Demographic studies indicate a dramatic global increase in the aging population over the past decade (He et al. 2016, Navaneetham and Arunachalam 2022). As life expectancy continues to rise—driven by advances in healthcare and socioeconomic conditions—promoting healthy aging has become a critical societal concern. The average age of the working population is also increasing, underscoring the need for interventions that especially preserve cognitive function in older adults (Tsai et al. 2024). Therefore, identifying effective strategies to support and maintain cognitive capacities during aging represents a vital and timely focus in current neuroscience research.

Dynamic changes in neural activity provide deeper insights into the complex mechanisms underlying cognitive decline in the aging brain, particularly through studies utilizing aging rodent models. Age-related alterations in electroencephalographic (EEG) activity commonly include reductions in alpha power, a shift toward slower dominant frequencies, and increased theta and delta activity (Ishii et al. 2017). These changes are indicative of global “slowing” of EEG rhythms in relation to impaired synaptic plasticity, and progressive neurodegeneration. Importantly, such oscillatory patterns have been closely linked to cognitive deficits observed in elderly individuals (Stomrud et al. 2010). Moreover, alterations in sleep EEG architecture may offer further understanding of age-related cognitive decline, as sleep plays a crucial role in memory consolidation and neural restoration (Borbély et al. 1981, Girardeau and Lopes-Dos-Santos 2021, Klinzing et al. 2019).

However, traditional linear analyses of neural activity often fail to capture the nonlinear and nonstationary nature of EEG signals, potentially overlooking critical features of aging-related brain dynamics (Zappasodi et al. 2015). In this context, nonlinear approaches such as recurrence quantification analysis (RQA) may provide a novel framework for characterizing age-associated changes in neural complexity. RQA quantifies the recurrence patterns within EEG time series and provides metrics such as determinism (DET), entropy (ENTR), and laminarity (LAM) (Pitsik 2025). These measures offer deeper insights into the temporal organization, regularity, and complexity of brain activity, allowing for a more comprehensive assessment of the aging brain’s functional dynamics.

In the present study, a rodent model combining D-galactose administration with urethane anesthesia was employed to investigate aging-related alterations in sleep architecture using RQA parameters. D-galactose is widely utilized to induce oxidative stress and simulate key features of aging at both cellular and behavioral levels (Cui et al. 2006, Kumar et al. 2010). Urethane, a long-acting general anesthetic, is known to produce EEG patterns that closely resemble natural sleep, particularly slow-wave and REM-like states (Clement et al. 2008), although recent studies also showed diverse mechanisms especially in slow network oscillations. Besides, both rats and humans exhibit cortical theta wave activity during REM sleep and delta activity during slow wave sleep (Kantar-Gok et al. 2017, Karlsson et al. 2022). This model allows for the controlled examination of age-associated neural dynamics, offering a valuable platform for exploring the nonlinear characteristics of sleep-related brain activity through RQA metrics.

In recent years, the exploration of neuroprotective compounds derived from natural sources has gained considerable attention as potential therapeutic agents. Among these, rosmarinic acid (RA)—a naturally occurring polyphenol found in culinary herbs such as rosemary (Rosmarinus officinalis) and basil (Ocimum basilicum)—has demonstrated potent antioxidant, anti-inflammatory, and cholinergic-modulatory properties, all of which might be critical for preserving neuronal function during aging (Alkam et al. 2007, Noguchi-Shinohara et al. 2020). Experimental studies utilizing various rodent models of neurodegeneration have demonstrated that RA administration can alleviate cognitive impairments and reduce oxidative stress (Kantar-Gok et al. 2017, Kantar Gok et al. 2018). RA has been shown to restore mismatch negativity in Alzheimer’s disease (AD) model rats and protect against oscillatory alterations associated with ovariectomy (OVX) (Kantar-Gok et al. 2017, Kantar Gok et al. 2018). In addition, RA has been reported to enhance non-REM sleep in humans and rats, as indicated by increased delta-wave power and decreased alpha-wave power in EEG recordings (Kwon et al. 2017, Morde et al. 2023, Tubbs and Randomized 2021). However, to date, no direct EEG studies in aged animals or humans have investigated RA’s impact on age-related nonlinear dynamics—such as entropy—which reflect neural complexity. While previous EEG studies have investigated aspects of RA’s neuroprotective effects, this study specifically applies RQA to evaluate how RA influences age-related changes in cortical complexity, as proof of concept. Consequently, further investigation is warranted to validate RA’s modulatory impact, particularly in the context of aging.

## Methods

### EEG dataset

This study retrospectively focused on the analysis of EEG recordings using the RQA method; therefore, different EEG recordings were combined and standardized to create an EEG database. Detailed information on the experimental models related to the included recordings is provided in the relevant references (Kantar-Gok et al. 2017, Karlsson et al. 2022). Ethical approval was additionally obtained for this study (Approval no: 2025.06.B.004). Rats (female, weighing 250–300 g, Wistar rats, aged three months) were randomly divided into four groups: Group 1: rats were sham operated and treated with saline (i.p. and gavage) (S, *n* = 18); Group 2: rats were sham operated and treated with saline (i.p.) and RA (gavage) (R, *n* = 18); Group 3: rats were treated with D-galactose (i.p.) and saline (gavage) (DG, *n* = 18); Group 4: rats were treated concomitantly with D-galactose (i.p.) and RA (gavage) (DGR, *n* = 18). D-Galactose (80 mg/kg/day) was administered by i. p. injection and RA (50 mg/kg/day) was given via gavage for 60 days. All experiments were approved by the Akdeniz University Animal Care and Use Committee and were performed in accordance with the European Community directive.

### EEG data acquisition

EEG was recorded between 09:00 am and 02:00 p.m. Rats were anesthetized (24 g/100 ml) with intraperitoneal injections of urethane (1.2 g/kg, Sigma-Aldrich, St Louis, MO, USA). The head of the anesthetized animal was attached to the standard stereotaxic frame, and four small holes (1.5 mm diameter) were drilled for the placement of the stainless-steel electrodes. Recording electrodes were placed bilaterally on temporal (AP:−4.5 mm, ML:−3.5 and + 3.5 mm) and frontal cortices (AP:+5 mm, ML:−2 and +2 mm) and reference and ground electrodes were placed on the cerebellar skull. The anesthetized animal was moved into a sound-attenuated recording room. The EEG signal was amplified (Brainamp EEG/EP Amplifier, Brain Products, Munich, Germany), band- pass filtered (0.1–300 Hz), and digitized at a 1000 Hz sampling rate (Brainvision Recorder, Brain Products, Munich, Germany). EEG signal was recorded for 10 min.

### EEG data preprocessing

The EEG data were filtered (0.1–150 Hz) and each period was segmented into 2 s epochs. Frequency analysis was performed using a fast Fourier transform (FFT) algorithm with a 10% Hanning window and spectral EEG powers computed. The EEG variables chosen were absolute power in two frequency bands, delta (0.5–3.5 Hz), theta (4–8 Hz). Filtering and segmentation were performed with the BrainVision Analyzer program (Brain Products GmBH). Each EEG epoch exported to MATLAB and all RQA analyses were performed in MATLAB.

### RQA analysis

Since its introduction by Eckmann et al., RQA has been widely utilized as a nonlinear analytical method for understanding the dynamics of non-periodic time series (Eckmann et al. 1987). Owing to the inherently non-linear and non-stationary characteristics of EEG signals, RQA has become a valuable tool for identifying hidden patterns associated with physiological events and neurodynamic processes (Pitsik 2025).

In the present study, following the completion of standard preprocessing procedures, the EEG data were segmented into 2-second epochs, and RQA was applied specifically to the delta and theta frequency bands.

**Fig. 1:**
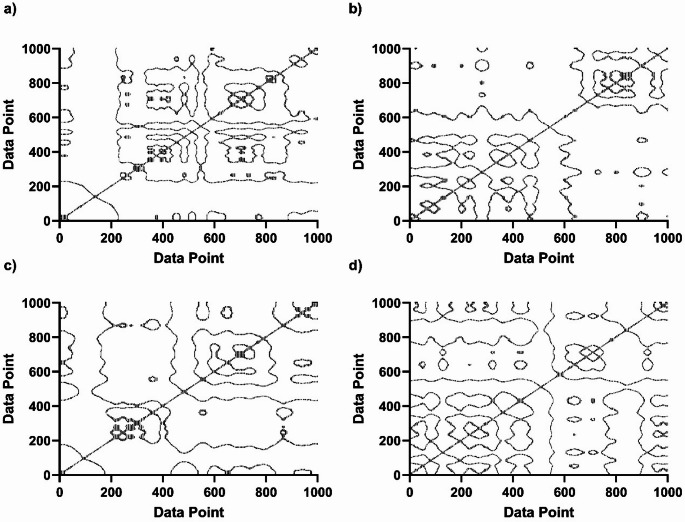
The representative recurrence plot examples for each group. Qualitative inspection of the recurrence plots reveals that aged animals (**c**) exhibit denser and more elongated diagonal and vertical structures compared to the sham group. These visual patterns represent the physical manifestation of increased DET and LAM, signaling a transition toward more predictable and rigid neural states in aging, which are visibly softened in the RA-treated group **d**. Both axes represent time indices of the EEG signal and that recurrence points indicate temporal similarity between system states. **a** rats were sham operated and treated with saline (i.p. and gavage) (sham) (S); **b** rats were sham operated and treated with saline (i.p.) and RA (gavage) (R); **c** rats were treated with D-galactose (i.p.) and saline (gavage) (DG); **d** rats were treated concomitantly with D-galactose (i.p.) and RA (gavage) (DGR)

All the RQA analysis were performed using custom MATLAB scripts implemented based on established algorithms described in the referenced (Marwan and Kurths 2002). In accordance with established practices reported in previous literature, the embedding dimension (m = 5), time delay (τ = 26 samples), and fixed recurrence rate (RR = 12.5%; lmin=vmin = 2) for RQA were optimized through average mutual information, false nearest neighbors screening and sensitivity analyses across all segments and then fixed for the study (Supplementary Fig. 1) (Bonnette et al. 2020, Riehm et al. 2024). Although τ = 26 samples correspond to a short temporal delay, the effective embedding window ((m − 1)τ ≈ 104 ms) spans a substantial portion of the theta cycle and a meaningful segment of the delta cycle. Thus, the analysis captures the continuous structural evolution (microdynamics) of band-limited oscillations within a phase-space trajectory, rather than treating signal values (data points) such as certain time like peak amplitude. This fixed thresholding approach was employed to mitigate common issues in RQA application, such as the need for individualized threshold estimation and the variability of recurrence density across epochs. By maintaining a constant RR, the comparability and interpretability of recurrence plots across different EEG segments were improved. The recurrence plot examples for each group are presented in Fig. 1.

Subsequently, key RQA-based features—Determinism (DET), Entropy (ENTR), and Laminarity (LAM)—were extracted from the recurrence plots. These features offer critical insights into the signal dynamics: DET reflects the degree of predictability or regularity within the signal; ENTR quantifies the complexity of the deterministic structures, capturing the richness of the pattern distributions; and LAM measures the proportion of recurrent points forming laminarity (vertical structures), which indicates the tendency of the system to remain in the same state over time.

The mathematical formulations of these parameters are defined as follows in the subsequent Eq. 

1 $$DET=\frac{{\mathop \sum \nolimits_{{l={l_{min}}}}^{N} lP\left( l \right)}}{{\mathop \sum \nolimits_{{l=1}}^{N} lP\left( l \right)}}$$

2 $$ENTR= - \mathop \sum \nolimits_{{l={l_{min}}}}^{N} p\left( l \right)lnp\left( l \right)$$

3 $$LAM=\frac{{\mathop \sum \nolimits_{{l={l_{min}}}}^{N} lP\left( l \right)}}{{\mathop \sum \nolimits_{{l=1}}^{N} lP\left( l \right)}}$$

where *l is the length of diagonal lines*,* N is the maximal line length*,* P(l)* is the number of diagonal line which length is *l*.

### Statistical analysis

Statistical analysis of data was performed by using one-way ANOVA and Tukey test (The Tukey test inherently corrects for multiple comparisons to control the family-wise error rate) were used for comparing subgroups by SPSS (SPSS 18.0, SPSS Inc., Chicago, IL) software for Windows. During comparison, values smaller than 0.05 (*p* < 0.05) were accepted significantly for all results. Data are represented as mean ± SEM.

## Results

### Delta waves RQA

**Fig. 2:**
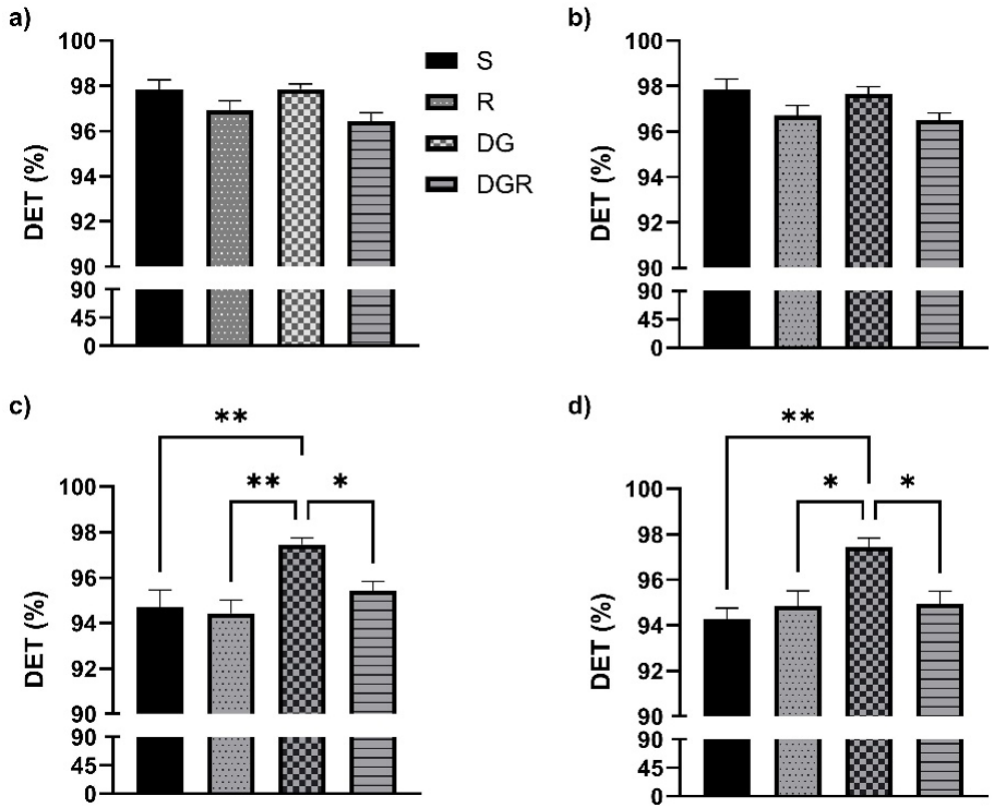
Determinism (DET) values derived from recurrence quantification analysis (RQA) in the delta frequency band, shown for frontal (**a**, **b**) and temporal cortices (**c**, **d**) across experimental groups. DET values remained statistically unchanged in the frontal cortex across groups, suggesting minimal impact of aging or rosmarinic acid (RA) in that region under urethane anesthesia. In contrast, the temporal cortex showed a significant increase in DET in the d galactose administration (DG) group compared to sham (S) (*p* < 0.05), indicating greater regularity and reduced variability in EEG patterns with aging. This increase was attenuated by RA administration (DGR), where DET values were significantly lower than in the DG group (*p* < 0.05), suggesting a partial restoration of signal flexibility. The RA-only group (R) did not differ from sham, supporting RA’s specificity for modulating aging-related rigidity. *, *p* < 0,05, **, *p* < 0,01

**Fig. 3:**
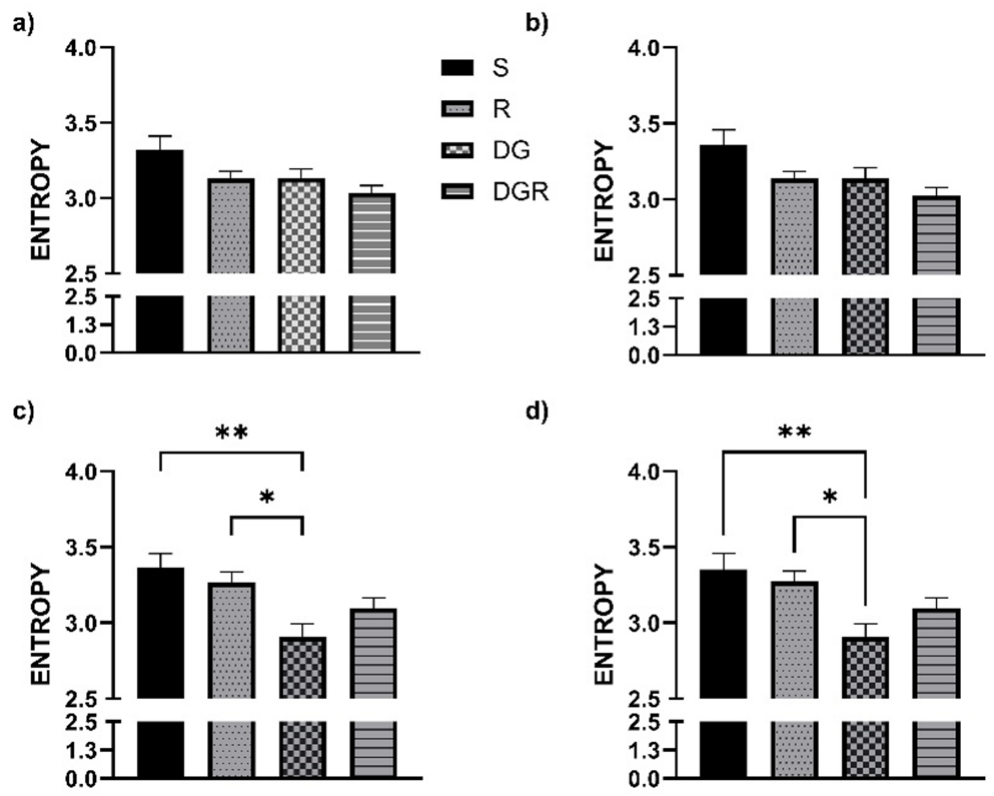
Entropy (ENTR) values across the delta band for frontal (**a**, **b**) and temporal (**c**, **d**) cortices. ENTR values, reflecting the complexity of deterministic patterns, were consistent across groups in the frontal cortex. However, in the temporal cortex, the d galactose+rosmarinic acid administration (DGR) group displayed significantly lower ENTR values than the sham and rosmarinic acid (RA) groups (*p* < 0.05), reflecting decreased signal complexity associated with aging. Although RA treatment in DGR rats led to a non-significant upward trend in ENTR compared to d galactose administration (DG), the difference did not reach statistical significance. This pattern suggests that aging diminishes neural entropy in the temporal region, and RA may exert a moderating effect. *, *p* < 0,05, **, *p* < 0,01

**Fig. 4:**
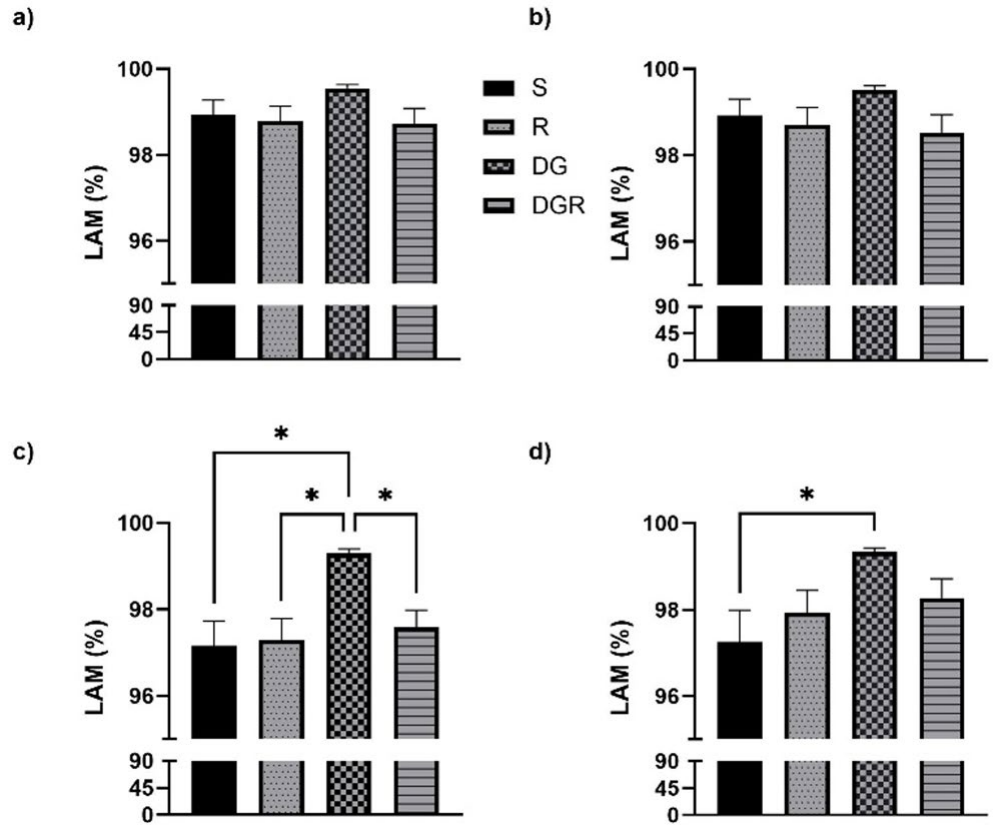
Laminarity (LAM) values in the delta frequency band for frontal (**a**, **b**) and temporal (**c**, **d**) cortices. LAM represents the proportion of vertical structures in recurrence plots, time interval where the state of the system does not change significantly. In frontal regions, LAM remained statistically similar across groups. In contrast, temporal cortex LAM values were significantly elevated in aged animals (DG) compared to sham (S) and RA-only (RA) groups (*p* < 0.05), consistent with enhanced signal rigidity and dwell time in singular dynamic states. D galactose+ rosmarinic acid administration (DGR) significantly reduced LAM compared to DG (*p* < 0.05), again indicating a potential reversal of age-induced neural persistence patterns. *, *p* < 0,05, **, *p* < 0,01

In the frontal regions, no significant changes in the DET parameter were observed following aging or RA administration, contrasting with the alterations detected in the temporal cortex. DET values for all groups, covering both frontal (Fig. 2a, b) and temporal areas (Fig. 2c, d), are summarized in Fig. 2. A slight but statistically significant reduction in DET was detected in both the RA and DGR groups. Regarding the ENTRP parameter, statistical analyses did not reveal any meaningful group differences. Nonetheless, in contrast to DET, ENTRP values remained relatively consistent across groups, except in the sham condition. These distributions are shown in Fig. 3, which illustrates data from both the frontal (Fig. 3a, b) and temporal regions (Fig. 3c, d). A similar trend was noted for the LAM parameter, which displayed strong concordance with DET outcomes. No statistically significant variations were identified among the experimental groups (*p* > 0.05). The corresponding LAM values for both cortical regions are presented in Fig. 4a, b.

In the temporal cortex, aging was associated with a pronounced increase in DET, reflecting greater signal regularity (Fig. 2c, d), and in LAM, indicating extended residence within the same dynamical state (Fig. 4c, d). These alterations were consistently observed across both hemispheres and reached statistical significance when compared with all other groups (*p* < 0.05). While the RA group did not significantly differ from either the sham or R groups, a notable divergence emerged when compared to the DGR group (*p* < 0.05 for both DET and LAM). This suggests that the increased rigidity and regularity characteristic of aging may be partially attenuated by RA treatment. Conversely, ENTRP values were significantly reduced in the DGR group relative to the sham and R groups (*p* < 0.05), reflecting a decrease in signal complexity associated with aging (Fig. 3c, d). Although RA administration elicited a slight upward trend in ENTRP, this change did not reach statistical significance when comparing the DG and DGR groups (*p* > 0.05).

### Theta waves RQA

Theta waves have increasingly become a focus of investigation in the context of physiological aging, establishing themselves as a key EEG component in recent research (Karlsson et al. 2022, Chaves-Coira et al. 2023). Therefore, similar to the approach taken with delta waves, the study examined theta activity using RQA, specifically evaluating the DET, ENTRP, and LAM parameters.

**Fig. 5:**
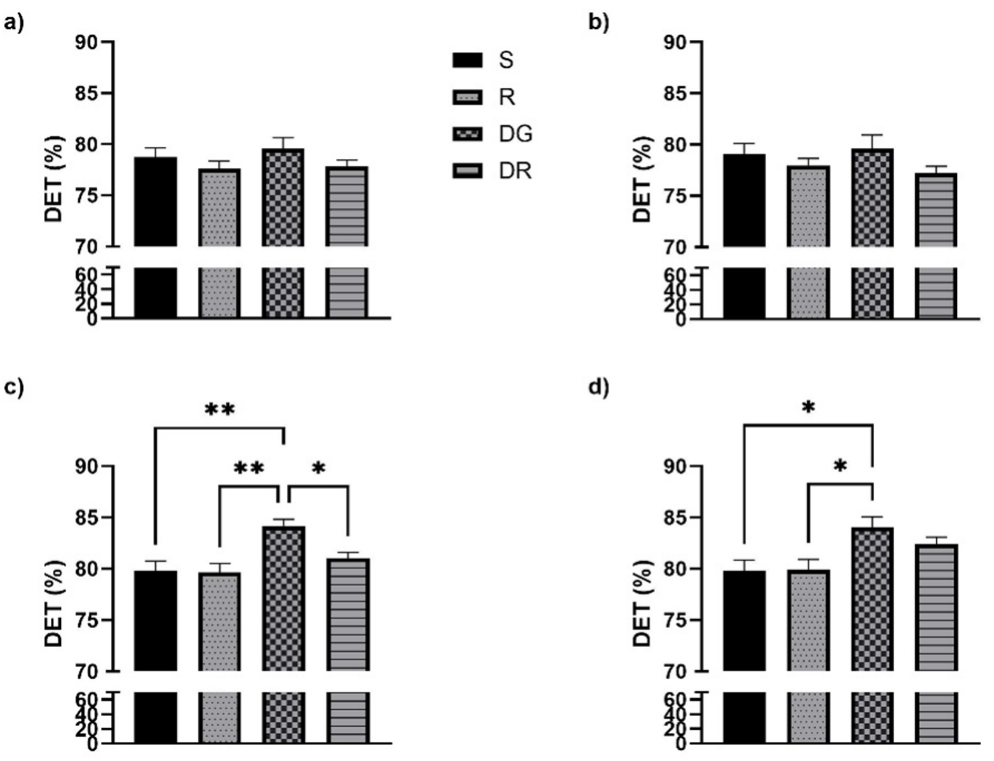
Determinism (DET) results in the theta frequency band for frontal (**a**, **b**) and temporal cortices (**c**, **d**). Theta-DET values did not significantly differ between groups in frontal regions, mirroring the delta-band results. However, in the temporal cortex, DET was significantly increased in the d galactose administration (DG) group (*p* < 0.05), reflecting higher determinism in aging. RA-treated animals (DGR) showed lower DET values in the right hemisphere compared to DG (*p* < 0.05), but not in the left, indicating a potential lateralized modulation by rosmarinic acid (RA). These results align with delta band findings and support the temporal cortex as a primary site of aging-induced rigidity in EEG dynamics. *, *p* < 0,05, **, *p* < 0,01

**Fig. 6:**
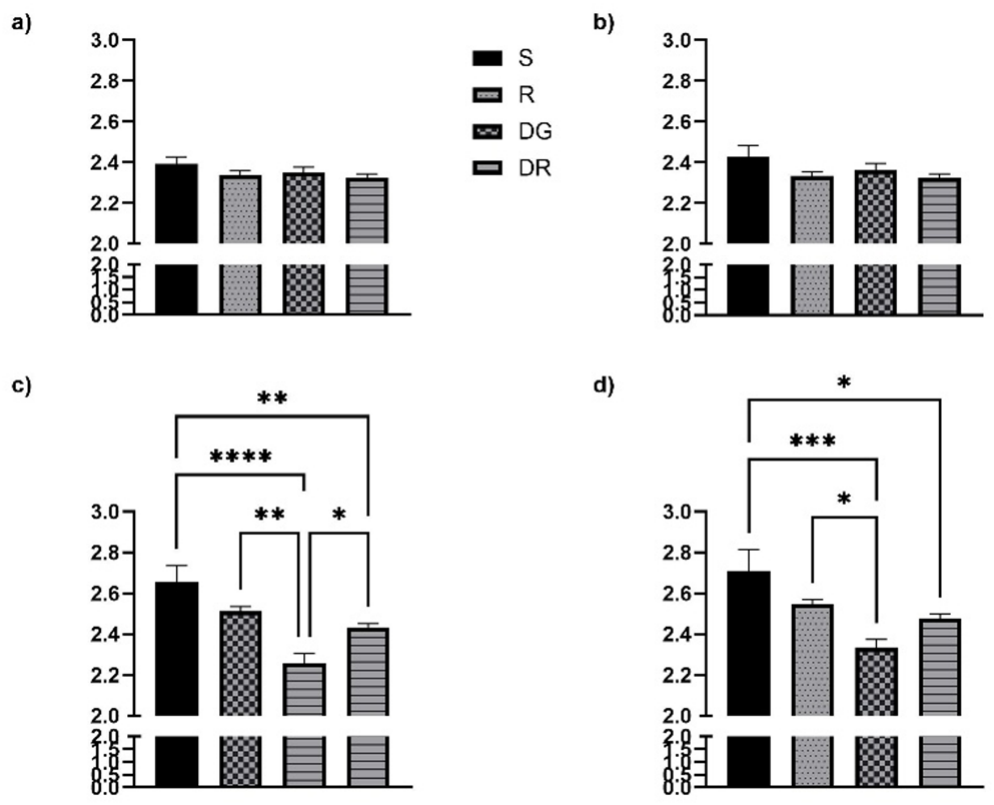
Entropy (ENTR) values in theta frequency band for frontal (**a**, **b**) and temporal cortices (**c**, **d**). No group differences were detected in frontal cortex ENTR across groups. In the temporal cortex, ENTR was significantly reduced in the d galactose+ rosmarinic acid administration (DGR) group compared to sham (S), rosmarinic acid (RA), and d galactose administration (DG) groups in the right hemisphere (*p* < 0.05), suggesting that combined aging and RA treatment may impact the complexity of oscillatory patterns more than either condition alone. The left hemisphere showed a similar but non-significant trend. These findings highlight a possible asymmetric effect of RA on EEG complexity in aging. *, *p* < 0,05, **, *p* < 0,01

**Fig. 7:**
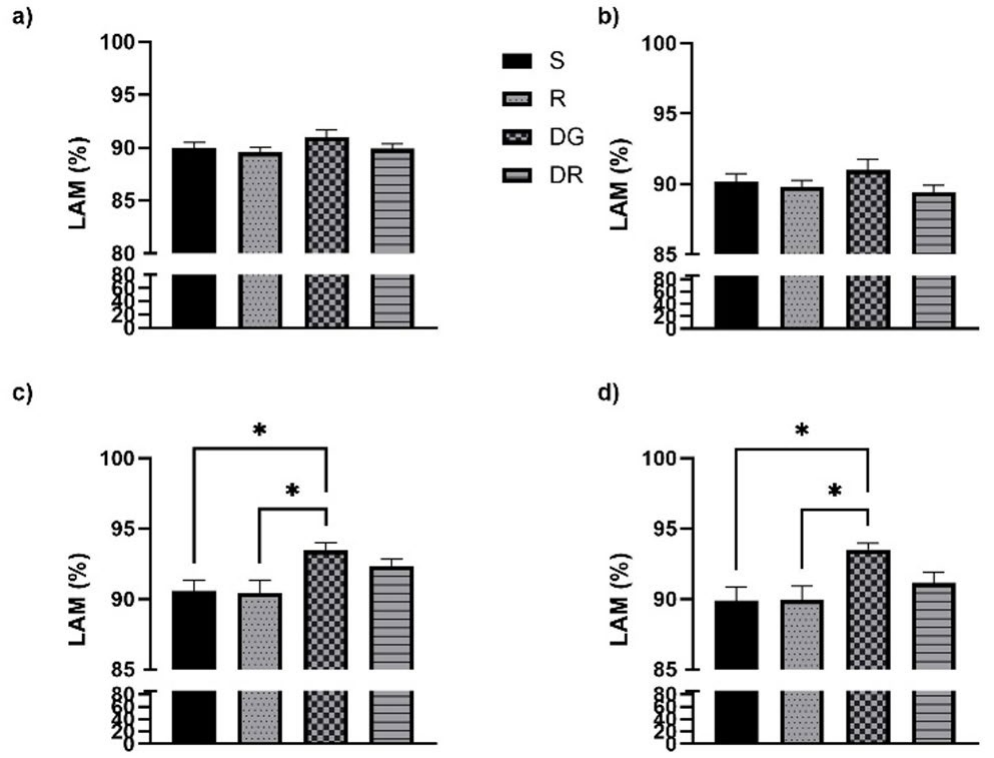
Laminarity (LAM) values in the theta frequency band for frontal (**a**, **b**) and temporal cortices (**c**, **d**). Temporal cortex LAM was significantly increased in d galactose administration (DG) rats relative to all other groups, particularly in the right hemisphere (*p* < 0.05), indicating stronger recurrence of persistent states in aged brains. Rosmarinic acid (RA) administration significantly reduced LAM in the right hemisphere compared to DG (*p* < 0.05), while the left hemisphere showed no significant group differences. These patterns suggest that aging increases neural persistence in the temporal cortex and that RA may partially restore temporal lability in a lateralized manner. *, *p* < 0,05, **, *p* < 0,01

As observed with delta activity, no statistically significant changes were detected in DET values in the frontal regions following aging or RA administration, in contrast to the findings in the temporal cortex. DET values for all experimental groups are summarized in Fig. 5, which includes both frontal (Fig. 5a, b) and temporal (Fig. 5c, d) regions. Statistical analysis of the ENTRP parameter did not reveal any significant group differences in the frontal cortex. Nevertheless, similar to the delta findings, ENTRP values appeared relatively consistent across all groups except the sham group. These distributions are presented in Fig. 6, which includes both frontal (Fig. 6a, b) and temporal (Fig. 6c, d) data. For the LAM parameter, a pattern closely aligned with DET was observed, again demonstrating high concordance between the two measures. No statistically significant differences were identified between the experimental groups (*p* > 0.05). The corresponding LAM values for both cortical regions are illustrated in Fig. 7a, b.

In the temporal cortex, aging was associated with a significant increase in both DET (Fig. 6c, d) and LAM (Fig. 7c, d). However, RA administration did not produce a statistically significant change in DET values in the left hemisphere when compared to the DGR group (*p* > 0.05), although a significant difference was observed in the right hemisphere between the DG and DGR groups (*p* < 0.05). A similar trend was observed for the LAM parameter: while a statistically significant difference emerged between the DG and DGR groups in the right hemisphere (*p* < 0.05), no such difference was found in the left hemisphere between DG and either the R or DGR groups (*p* > 0.05). Given that DET and LAM values were similar across hemispheres within the DG group, this pattern may suggest a potential effect of RA in modulating age-related changes. In contrast, ENTRP values in the right hemisphere showed a significant reduction in the DGR group when compared to the sham, R, and DG groups (*p* < 0.05). Moreover, a statistically significant difference was also found between the sham and DGR groups (*p* < 0.05). This may reflect that aging has a greater impact on theta oscillations than on delta waves in the temporal cortex, and that RA treatment may be insufficient to reverse this reduction in complexity.

In the left hemisphere, similar to DET and LAM findings, no statistically significant difference was observed between the DG and DGR groups. However, a significant reduction in ENTRP was noted when comparing the DGR group to the sham group (*p* < 0.05). To further explore potential hemispheric asymmetries, statistical comparisons were made between the two hemispheres within each group. These analyses did not yield statistically significant differences (*p* > 0.05), suggesting that although mean values across hemispheres were close, the observed variability may be attributed to differences in SEM values between hemispheres.

## Discussion

This study explores age-related alterations in EEG frequency bands under urethane anesthesia as a known model for sleep through nonlinear analytical approaches and examines the modulatory effects of RA as a therapeutic agent, presenting valuable findings that contribute to a deeper understanding of the sleep oscillatory architecture during aging. It is known that urethane anesthesia EEG has two phases in rats such as SWS and REM sleep which is similar to natural sleep. Firstly, the observed increase in DET within the temporal regions of aged animals indicates a more deterministic pattern in both sleep related delta and theta frequency bands with aging. Moreover, the strong alignment between the LAM parameter and DET suggests that this heightened deterministic structure is not only present but also frequently repeated, providing a quantitative indication of persistent neural rigidity. The concurrent decrease in ENTRP further supports the dominance of this rigid pattern within the aging model, highlighting a general reduction in neural complexity. In view of well documented role of sleep oscillatory patterns in memory consolidation, these findings also demonstrate that RA has the potential to modulate these parameters significantly, underscoring its promise as a therapeutic compound capable of altering age-related changes in brain dynamics.

Physiological aging has long been a major topic of investigation from both structural and functional perspectives. Functionally, many studies exploring physiological aging have focused on identifying how brain activity changes through EEG recordings. In addition, findings such as a diminished dynamic response to external stimuli and the characterization of aging as a “loss of complexity” have underscored the need for nonlinear methods alongside conventional analyses in EEG-based aging research (Takahashi et al. 2009). Previous studies have consistently reported a reduction in the amplitude of alpha activity and a general slowing of dominant alpha rhythms in aging (Ishii et al. 2017). However, it remains challenging to reach a consensus regarding other EEG oscillations such as delta and theta patterns (Rossini et al. 2006, Rossini et al. 2007). Although increased theta band power has been reported in posterior regions in some studies during rest, the lack of consensus highlights the need for continued research in this area (Stomrud et al. 2010, Cummins and Finnigan 2007, Vlahou et al. 2014). Sleep EEG oscillations are a complementary approach to understanding the neuronal correlate of aging process. SWS sleep EEG delta power reflects neural plasticity and, in line with age-related cognitive decline decreases with age (Kattler et al. 1994). Alterations in sleep EEG oscillations were repeatedly hypothesized to play a crucial role in cognitive aging (Dijk et al. 1989, Fogel et al. 2012), with decreasing slow wave activity arguably being the most Important age-related change in the sleep EEG. Conversely, age-related increases of power in frequency bands indicating brain arousal have been reported during SWS sleep stages. Thus, these age-related architectural changes indicate a generalized “lightening” of sleep due to reduced homeostatic sleep drive or ability to respond to sleep drive. The growing acceptance of the “loss of complexity” or “decrease in complexity” hypothesis in physiological aging has led to increased use of entropic approaches in EEG analysis (Zappasodi et al. 2015, Lipsitz and Goldberger 1992). Our findings indicate that sleep related entropy significantly decreases in the temporal cortex within the aging model. Considering the region’s close connection with subcortical structures, this suggests a reduction in input variability and the emergence of increasingly rigid signal patterns. These results are also consistent with the observed increase in DET and LAM parameters. While DET reflects a more deterministic signal organization, LAM is indicative of repetitive structures. Together, they support the idea that aging involves decreased input variability and more frequent recurrence of similar patterns—pointing to a decline in functional connectivity between brain networks (Grady et al. 2010) which may lead to consolidation deficiency and metabolic failure. Moreover, our data reveal that RA administration significantly modulates these parameters (DET, ENTRP and LAM), especially in the temporal regions. This therapeutic effect may arise via multiple mechanisms. First, RA has previously been identified as a natural peroxynitrite scavenger (Alkam et al. 2007). Peroxynitrite, in turn, has been implicated not only in pathological processes but also as a key contributor to physiological aging (Huang et al. 2023, Maruyama et al. 2001), highlighting the antioxidant potential of RA. A second important mechanism involves RA’s capacity to increase acetylcholine levels and influence the cholinergic system (Kantar et al. 2022). It is well known that age-related declines in cholinergic function impair cortical activity (Chaves-Coira et al. 2023). The fact that physostigmine—a cholinesterase inhibitor—can reverse such age-related damage further underscores the importance of the cholinergic system in aging. Taken together, these findings suggest that RA’s benefits may extend beyond its antioxidant action and include neuromodulation via cholinergic pathways. Moreover, in a placebo-controlled clinical trial, although no significant differences were observed in cognitive measurements among participants receiving RA, improvements in Neuropsychiatric Inventory Questionnaire scores were reported—findings that align with the temporal region-specific changes observed in our study (Noguchi-Shinohara et al. 2020).

Notably, no significant changes were observed in the frontal regions in our study. However, Zappasodi et al. previously reported decrease in EEG complexity in frontal regions, aligning it explicitly with the “loss of complexity” framework using fractal dimension analysis which is another well-known non-linear analysis method (Zappasodi et al. 2015). The absence of similar findings in our data may stem from the use of anesthetized rats, as EEG recordings under anesthesia are known to exhibit increased frontal power (Jäntti and Sloan 2008, John and Prichep 2005). The high DET values in the frontal region across all groups, which exceeded those of the temporal regions, are consistent with this explanation. Additionally, the concept of “loss of complexity” is considered an early sign of aging in the temporal cortex, and our model may be better suited to highlighting this early-stage phenomenon.

In conclusion, this study employed RQA, a nonlinear method, to examine the impact of physiological aging on EEG frequency bands. The results quantitatively demonstrate that aging leads to the emergence of more rigid and repetitive structures, particularly in lower frequency bands. Furthermore, the finding that RA administration can reverse these alterations—especially in the temporal cortex—suggests that RA may serve as a promising therapeutic agent in physiological aging. To our knowledge, this is the first study to investigate cortical electrophysiological responses and the effects of RA during aging using RQA, a technique increasingly recognized for its ability to capture the nonlinear dynamics of EEG. Furthermore, our findings—specifically the observed increase in rigid patterns within delta and theta bands in temporal regions—contribute to the field by providing a dynamical perspective that may explain why power-based measurements are inconsistent. RQA reveals that even if power levels remain stable, the underlying ‘flexibility’ and complexity of these oscillations decline with age, offering a more sensitive metric for neurodegeneration than spectral power alone. While traditional linear analyses have failed to reach a consensus, our findings suggest that the fundamental change in aging is not merely a shift in power, but a transformation in dynamical structure. Specifically, the significant increase in DET and LAM, coupled with the decline in ENTR, indicates that theta oscillations in the aged brain transition toward a more deterministic and rigid state. By focusing on the temporal organization of the signal rather than its energy distribution, RQA effectively reflects age-related neural dynamics in the EEG. It should also be acknowledged that one limitation of this study is the use of EEG recordings obtained under anesthesia, which may have masked potential differences in the frontal regions.

## Supplementary Information

Below is the link to the electronic supplementary material.

Supplementary Material 1

## Data Availability

Data will be made available on request.

## References

[CR1] Alkam T et al (2007) A natural scavenger of peroxynitrites, rosmarinic acid, protects against impairment of memory induced by Abeta(25–35). Behav Brain Res 180(2):139–145 17420060 10.1016/j.bbr.2007.03.001

[CR2] Bonnette S et al (2020) Electrocortical dynamics differentiate athletes exhibiting low- and high- ACL injury risk biomechanics. Psychophysiology 57(4):e13530 31957903 10.1111/psyp.13530 PMC9892802

[CR3] Borbély AA et al (1981) Sleep deprivation: effect on sleep stages and EEG power density in man. Electroencephalogr Clin Neurophysiol 51(5):483–495 6165548 10.1016/0013-4694(81)90225-x

[CR4] Chaves-Coira I et al (2023) Cognitive Deficits in Aging Related to Changes in Basal Forebrain Neuronal Activity. Cells 12(11):1477 37296598 10.3390/cells12111477 PMC10252596

[CR5] Clement EA et al (2008) Cyclic and sleep-like spontaneous alternations of brain state under urethane anaesthesia. PLoS One 3(4):e2004 18414674 10.1371/journal.pone.0002004 PMC2289875

[CR6] Cui X et al (2006) Chronic systemic D-galactose exposure induces memory loss, neurodegeneration, and oxidative damage in mice: protective effects of R-alpha-lipoic acid. J Neurosci Res 83(8):1584–1590 16555301 10.1002/jnr.20845

[CR7] Cummins TDR, Finnigan S (2007) Theta power is reduced in healthy cognitive aging. Int J Psychophysiol 66(1):10–17 17582632 10.1016/j.ijpsycho.2007.05.008

[CR8] Dijk DJ, Beersma DG, van den Hoofdakker RH (1989) All night spectral analysis of EEG sleep in young adult and middle-aged male subjects. Neurobiol Aging 10(6):677–682 2628779 10.1016/0197-4580(89)90004-3

[CR9] Eckmann JP, Kamphorst SO, Ruelle D (1987) Recurrence Plots of Dynamical Systems. Europhys Lett 4(9):973

[CR10] Fogel S et al (2012) NREM Sleep Oscillations and Brain Plasticity in Aging. Front Neurol 3:176 23248614 10.3389/fneur.2012.00176 PMC3522106

[CR11] Girardeau G, Lopes-Dos-Santos V (2021) Brain neural patterns and the memory function of sleep. Science 374(6567):560–564 34709916 10.1126/science.abi8370 PMC7611961

[CR12] Grady CL et al (2010) A multivariate analysis of age-related differences in default mode and task-positive networks across multiple cognitive domains. Cereb Cortex 20(6):1432–1447 19789183 10.1093/cercor/bhp207 PMC3181214

[CR13] He W, Goodkind D, Kowal PR (2016) An aging world: 2015. United States Census Bureau, Washington, DC

[CR14] Huang P et al (2023) A therapeutic probe for detecting and inhibiting ONOO in senescent cells. J Mater Chem B 11(11):2389–2396 36853656 10.1039/d2tb02568j

[CR15] Ishii R et al (2017) Healthy and Pathological Brain Aging: From the Perspective of Oscillations, Functional Connectivity, and Signal Complexity. Neuropsychobiology 75(4):151–161 29466802 10.1159/000486870

[CR16] Jäntti V, Sloan TB (2008) EEG and anesthetic effects. Handb Clin Neurophysiol 8:77–93

[CR17] John ER, Prichep LS (2005) The anesthetic cascade: a theory of how anesthesia suppresses consciousness. Anesthesiology (Philadelphia) 102(2):447–471 10.1097/00000542-200502000-00030 15681963

[CR18] Kantar D, Acun AD (2022) Er H (2022) Evaluate the effects of rosmarinic acid in ovariectomized rats: urethane-induced cortical oscillations. Turk Bullet Hygiene Exp Biol/Türk Hijyen ve Deneysel Biyoloji 79(4):632–645

[CR19] Kantar Gok D et al (2018) Protective role of rosmarinic acid on amyloid beta 42-induced echoic memory decline: Implication of oxidative stress and cholinergic impairment. Neurochem Int 118:1–13 29655652 10.1016/j.neuint.2018.04.008

[CR20] Kantar-Gok D et al (2017) Changes of auditory event-related potentials in ovariectomized rats injected with d-galactose: Protective role of rosmarinic acid. Neurotoxicology 62:64–74 28501655 10.1016/j.neuro.2017.05.003

[CR21] Karlsson AE, Lindenberger U, Sander MC (2022) Out of Rhythm: Compromised Precision of Theta-Gamma Coupling Impairs Associative Memory in Old Age. J Neurosci 42(9):1752–1764 34996815 10.1523/JNEUROSCI.1678-21.2021 PMC8896557

[CR22] Kattler H, Dijk DJ, Borbély AA (1994) Effect of unilateral somatosensory stimulation prior to sleep on the sleep EEG in humans. J Sleep Res 3(3):159–164 10607121 10.1111/j.1365-2869.1994.tb00123.x

[CR23] Klinzing JG, Niethard N, Born J (2019) Mechanisms of systems memory consolidation during sleep. Nat Neurosci 22(10):1598–1610 31451802 10.1038/s41593-019-0467-3

[CR24] Kumar A, Prakash A, Dogra S (2010) Naringin alleviates cognitive impairment, mitochondrial dysfunction and oxidative stress induced by D-galactose in mice. Food Chem Toxicol 48(2):626–632 19941926 10.1016/j.fct.2009.11.043

[CR25] Kwon YO, Hong JT, Oh KW (2017) Rosmarinic acid potentiates pentobarbital-induced sleep behaviors and non-rapid eye movement (NREM) sleep through the activation of GABA(A)-ergic systems. Biomol Ther (Seoul) 25(2):105–111 27469144 10.4062/biomolther.2016.035 PMC5340534

[CR26] Lipsitz LA, Goldberger AL (1992) Loss of ‘complexity’ and aging. Potential applications of fractals and chaos theory to senescence. JAMA 267(13):1806–1809 1482430

[CR27] Maruyama W et al (2001) Peroxynitrite induces neuronal cell death in aging and age-associated disorders: A review. J Am Aging Assoc 24(1):11–18 23604871 10.1007/s11357-001-0002-8 PMC3455645

[CR28] Marwan N, Kurths J (2002) Nonlinear analysis of bivariate data with cross recurrence plots. Phys Lett A 302(5):299–307

[CR29] Morde A et al (2023) Rosmarinic acid-A potential therapeutic agent for sleep disorders. Int J Pharm Sci 3(1):1–6

[CR30] Navaneetham K, Arunachalam D (2022) Global Population Aging, 1950–2050. In: Handbook of Aging, Health and Public Policy: Perspectives from Asia. Springer Nature Singapore: Singapore. pp 1–18

[CR31] Noguchi-Shinohara M et al (2020) Safety and efficacy of Melissa officinalis extract containing rosmarinic acid in the prevention of Alzheimer’s disease progression. Sci Rep 10(1):18627 33122694 10.1038/s41598-020-73729-2 PMC7596544

[CR32] Pitsik E (2025) Recurrence quantification analysis and theta-band functional networks detect age-related changes in brain sensorimotor system: VR-based approach. Eur Phys J Special Top 234:4329-4335

[CR33] Riehm CD et al (2024) Corticomuscular cross-recurrence analysis reveals between-limb differences in motor control among individuals with ACL reconstruction. Exp Brain Res 242(2):355–365 38092900 10.1007/s00221-023-06751-1 PMC10872341

[CR34] Rossini PM et al (2006) Conversion from mild cognitive impairment to Alzheimer’s disease is predicted by sources and coherence of brain electroencephalography rhythms. Neuroscience 143(3):793–803 17049178 10.1016/j.neuroscience.2006.08.049

[CR35] Rossini PM et al (2007) Clinical neurophysiology of aging brain: from normal aging to neurodegeneration. Prog Neurobiol 83(6):375–400 17870229 10.1016/j.pneurobio.2007.07.010

[CR36] Stomrud E et al (2010) Slowing of EEG correlates with CSF biomarkers and reduced cognitive speed in elderly with normal cognition over 4 years. Neurobiol Aging 31(2):215–223 18462837 10.1016/j.neurobiolaging.2008.03.025

[CR37] Takahashi T et al (2009) Age-related variation in EEG complexity to photic stimulation: a multiscale entropy analysis. Clin Neurophysiol 120(3):476–483 19231279 10.1016/j.clinph.2008.12.043 PMC2880484

[CR38] Tsai YIP et al (2024) Digital interventions for healthy ageing and cognitive health in older adults: a systematic review of mixed method studies and meta-analysis. BMC Geriatr 24(1):217 38438870 10.1186/s12877-023-04617-3 PMC10910826

[CR39] Tubbs AS (2021) A Randomized, Double-Blind, Placebo-Controlled Trial of a Polyphenol Botanical Blend on Sleep and Daytime Functioning. Int J Environ Res Public Health 18(6):3044 33809544 10.3390/ijerph18063044 PMC8000032

[CR40] Vlahou EL et al (2014) Resting-state slow wave power, healthy aging and cognitive performance. Sci Rep 4(1):5101 24869503 10.1038/srep05101 PMC4037748

[CR41] Zappasodi F et al (2015) Age-Related Changes in Electroencephalographic Signal Complexity. PLoS ONE 10(11):e0141995 26536036 10.1371/journal.pone.0141995 PMC4633126

