# Supplementary material for: Rosmarinic acid mitigates age-related loss of EEG complexity in temporal cortex: a recurrence quantification analysis
Source: Cogn Neurodyn. 2026 May 7;20(1):87. doi: 10.1007/s11571-026-10457-9 (PMC13153318; doi:10.1007/s11571-026-10457-9)
Supplement: Supplementary file 1 — Supplementary Material 1 [file 11571_2026_10457_MOESM1_ESM.docx]

**Supp. Fig. 1** Average mutual information (AMI) plotted as a function of time delay (τ, in samples). AMI values show a monotonic decrease with increasing time delay, reflecting strong temporal correlations in the signal. The time delay (τ = 26) samples was selected at the onset of the AMI curve's plateau following its initial local minimum.

**Supp. Fig. 2** False nearest neighbor (FNN) analysis used to determine the optimal embedding dimension for recurrence quantification analysis. The ratio of FNN is shown as a function of embedding dimension (m) using a fixed time delay of τ = 26 samples. FNN ratio decreases rapidly with increasing embedding dimension and reaches a stable plateau below 5% at m ≥ 5, indicating that an embedding dimension of m = 5 is sufficient to reliably reconstruct the underlying system dynamics.
